# The impact of sexual hormones on depressive symptoms and alcohol use in a woman with delusional disorder at the perimenopausal stage: case report and review of recent works

**DOI:** 10.1192/j.eurpsy.2022.2238

**Published:** 2022-09-01

**Authors:** G.F. Fucho, L. Delgado, A. González-Rodríguez, A. Guàrdia, A. Álvarez, S. Crivillés, J.D. Barbero, J.A. Monreal, J. Labad, D. Palao Vidal

**Affiliations:** 1 Parc Taulí University Hospital, Department Of Mental Health, Sabadell, Barcelona, Spain; 2 Mutua Terrassa University Hospital, Department Of Mental Health, Terrassa, Spain; 3 School of Medicine, Universitat Autònoma de Barcelona, Medicine, Cerdanyola del Vallés, Spain; 4 Centro de Investigación Biomédica en Red de Salud Mental (CIBERSAM), Salud Mental, Madrid, Spain; 5 Hospital of Mataró, Consorci Sanitari Del Maresme, Mataró, Spain

**Keywords:** Delusional disorder, Depression, Alcohol use, sexual hormones

## Abstract

**Introduction:**

The influence of sexual hormones on mental disorders have been extensively reported. In fact, recent studies suggest that sex hormones may play a relevant role in the pathophysiology of psychosis, may be a precipitant when exogenously administered or even be used as a treatment agent of psychotic disorders.

**Objectives:**

To describe the case of a patient with a recent diagnosis of delusional disorder (DD) with an onset in the perimenopausal period.

**Methods:**

Case report and narrative review focused on the impact of sexual hormones on depressive symptoms and alcohol use comorbidity during perimenopause in DD women by using PubMed database.

**Results:**

Case report: A 48-year-old woman diagnosed with DD. The clinical assessment of climacteric symptoms, as well as the gonadotropins (elevated levels of follicle-stimulating hormone -FSH- and luteinizing hormone -LH-) and estrogen levels monitoring (variations on 17-β-estradiol -E2-) enabled to link the endocrine changes with the onset and course of the psychiatric disorder. During the development of the disease, the patient also presented comorbid depressive symptoms and alcohol use disorder. Review: Estrogen depletion seems to increase the risk of psychosis, while scientific literature is not conclusive in establishing a definitive relationship between depressive symptoms and hormonal imbalance in DD. Alcohol use disorder is a common comorbidity in both perimenopausal women and patients with DD.

**Conclusions:**

Multiaxial management of the case helped clinicians to achieve clinical stabilization. Sex differences as well as hormonal pattern disturbances should receive special attention due to the seminal implications in pharmacotherapy and clinical outcomes.

**Disclosure:**

No significant relationships.

